# Continuous parallel ESI-MS analysis of reactions carried out in a bespoke 3D printed device

**DOI:** 10.3762/bjnano.4.31

**Published:** 2013-04-29

**Authors:** Jennifer S Mathieson, Mali H Rosnes, Victor Sans, Philip J Kitson, Leroy Cronin

**Affiliations:** 1 School of Chemistry, University of Glasgow, G12 8QQ, United Kingdom

**Keywords:** continuous parallel analysis, ESI-MS, 3D printing, reactionware, supramolecular chemistry

## Abstract

Herein, we present an approach for the rapid, straightforward and economical preparation of a tailored reactor device using three-dimensional (3D) printing, which can be directly linked to a high-resolution electrospray ionisation mass spectrometer (ESI-MS) for real-time, in-line observations. To highlight the potential of the setup, supramolecular coordination chemistry was carried out in the device, with the product of the reactions being recorded continuously and in parallel by ESI-MS. Utilising in-house-programmed computer control, the reactant flow rates and order were carefully controlled and varied, with the changes in the pump inlets being mirrored by the recorded ESI-MS spectra.

## Introduction

Flow chemistry is a growing field that can increase productivity and control, ensure reproducibility and reduce manual handling [1]. There is currently a huge interest in directly interfacing milli- and microfluidic reactor devices with analytical techniques, for high-throughput analysis with efficient structure elucidation and identification of compounds [2]. This type of interfacing offers a convenient method to monitor, in real-time, a dynamic process under flow “without unnecessary interruptions and sample manipulations” [3].

Two types of monitoring techniques for this type of chemistry, where no manual transfer of sample is required, are in-line and on-line, where in-line analysis refers to continuous analyses of the product stream, whilst on-line analysis refers to analyses of selected aliquots in a parallel stream [1]. Examples of techniques used for in-line, or on chip, analyses are UV–vis [4–6], IR [4,7–9], fluorescence [10], Raman [11] and NMR [12] spectroscopy. For on-line analysis, LC-MS or HPLC have typically been used, where fractions/batches of the sample-flow are directed into the analytical technique of choice [13–14]. Results from both types of techniques can be used to check if a reaction has gone to completion and if there are impurities or unwanted side-products. This data can also subsequently be used to change the reaction conditions to increase the yield and purity.

Electrospray ionisation mass spectrometry (ESI-MS) is an excellent analytical tool for flow chemistry due to its high sensitivity, allowing for low sample concentrations and production of structural information that you may not get from other techniques. There are several reports in which ESI-MS has been coupled to microfluidic devices as an in-line analytical technique [15–17]. In these instances all the flow was fed directly to the MS to ensure high enough flow and concentration for efficient detection, but whereby the sample cannot be regenerated due to the nature of MS. Examples also include fluidic devices coupled to on-line ESI-MS, for example to observe and analyse microdroplets from a microreactor [18]. More recently, Ley et al. reported a continuous flow reaction, which was monitored by on-line ESI-MS [1], in which a six-way valve was used to send aliquots directly to the MS for analysis. Although there are numerous reports on the use of MS as both an in-line and an on-line analytical technique, there are still limitations to overcome, with the main one being the inability to follow reactions in real time, under continuous flow, by in-line MS, whilst also collecting the product/outcome.

Traditionally, when interfacing flow devices with ESI-MS analysis complicated and expensive microscale fluidic devices have been required. Herein, we present an approach interfacing ESI-MS with a 3D-printed milliscale device, or tailored “reactionware” [4]. The use of 3D printing bypasses sophisticated manufacturing centres and promises to revolutionise every aspect of the way that materials are turned into functional devices, from design through to operation [19–24], with 3D printing producing bespoke, low-cost appliances that previously would have required dedicated facilities. 3D printing is a cheap chemical discovery tool, whereby the setup can cost thousands of pounds instead of tens of thousands (see Supporting Information File 1 for details), and which combines the disciplines of synthetic chemistry and chemical engineering in a reconﬁgurable and highly accessible format. The use of freely available CAD software, such as Autodesk123D^®^, and the rapid fabrication that comes with 3D printing, allows for the design and production of specific devices tailored to the intended chemical reaction. The high surface-area-to-volume ratio, precise control of volume, and manipulation of reaction environment results in strict control of the final device and the subsequent reactions carried out.

**Scheme 1:**
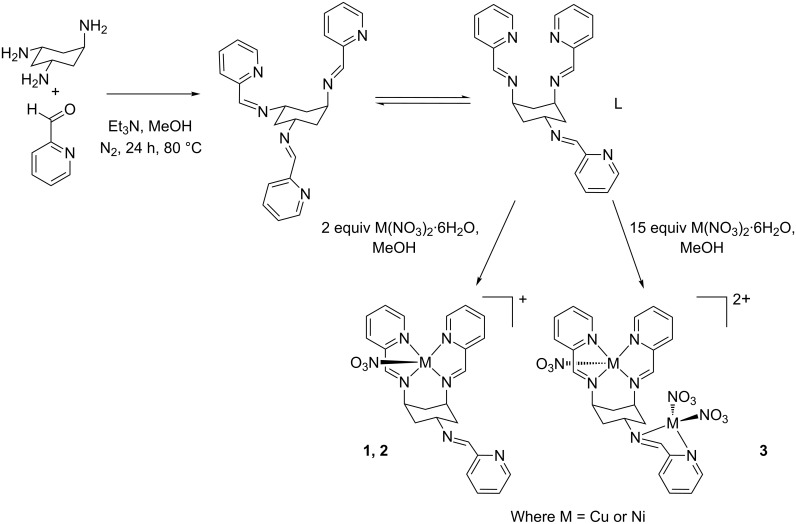
Reaction scheme for the formation of the ttop and metal-salt coordination complex (**1** is [Cu(C_24_H_24_N_6_)(NO_3_)]^+^; **2** is [Ni(C_24_H_24_N_6_)(NO_3_)]^+^; **3** is [Cu_2_(C_24_H_24_N_6_)(NO_3_)_2_]^2+^).

We have previously demonstrated the versatility and configurability of reusable and bespoke reactionware, in which a 3D-printed “reactionware” matrix, with the reagents printed directly into the device, was used to initiate chemical reactions [6]. We have also presented how 3D printing can be used to make intricate micro- and milliscale reactionware for organic, inorganic and materials syntheses, offering significant freedom to design bespoke reactors in terms of residence time, mixing points, inlets and outlets, et cetera [4]. Herein, we show that we can carry out complex supramolecular chemistry in milliscale reactionware, in which *cis,trans*-1,3,5-tris(pyridine-2-ylmethylene)cyclohexane-1,3,5-triamine (ttop) forms complexes with a number of metals, such as copper(II) and nickel(II), with the Cu complex ion previously observed by ESI-MS (see Scheme 1) [25]. The ttop and metal-salt solutions are introduced to the device through pumps, via the device inlets, and by changing the pump speed (hence altering the flow rate and reaction conditions inside the device), using an in-house-programmed computer controller, the resulting complexation reaction can be observed with in-line ESI-MS. Because the product stream is split after the device, a section of the stream flows directly into the ESI-MS, continuously and in real time, whilst the other section of the stream is collected. To our knowledge, this is the first example of a configurable, real-time, continuous parallel-flow technique using ESI-MS as an analytical tool with a 3D-printed device as the reaction vessel.

## Experimental

### 3D printing

**Figure 1:**
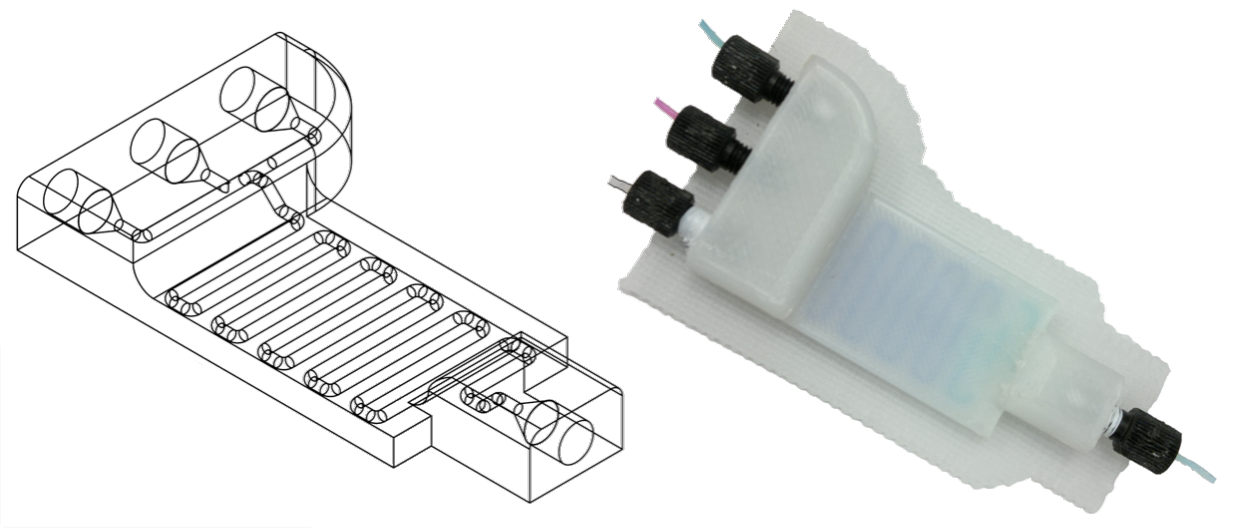
On the left is a schematic presentation of the STL file, whilst on the right is the device with screw fittings and connected with 1/16 inch (1.6 mm) tubing. Methylene blue and rhodamine B are being pumped through the device, which allows the inner-tube path to be rendered visible. A section consisting of only methylene blue can be seen at the front, followed by a stronger purple band, which is obtained from the successful mixing of rhodamine B and methylene blue.

Our device was designed using a freely distributed CAD software package (Autodesk123D^®^). The device design was exported as an STL file (see Supporting Information File 2), which was then interpreted by the Bits from Bytes Axon 2 software, which produces a 3D printer instruction file (BFB file), which was subsequently transferred to the 3DTouch^TM^ 3D printer. The printing was conducted in a layer-by-layer fashion, and the device used was printed in polypropylene (PP) by using a 3DTouch^TM^ printer, which deposits layers of thermopolymers through heated extruder nozzles to build the designed reactionware. PP is an attractive material for the fabrication of micro- and milliscale reactionware as it is an affordable, robust, flexible and relatively chemically inert polymer. The device designed and used for the work presented herein is shown in Figure 1 and consists of three inlets and one outlet. The overall dimension of the device is 46.5 × 80 mm, with an internal path diameter of 1.5 mm. The total internal volume of the device is roughly 0.65 mL, and the reaction volume is about 0.57 mL.

The inlets and outlets are designed to match the size of standard available screw fittings, which allows for easy connection to syringe pumps. The screw fittings are made of polyether ether ketone (PEEK), which is a harder plastic than PP, allowing for boring into the PP plastic inlet and outlets to give a tight seal to the device. These fittings can be removed and replaced as many times as necessary, and the whole device can be used repeatedly, for a variety of chemical reactions. LabView^®^ (Laboratory virtual instrumentation engineering workbench, a system design platform and development environment for a visual programming language from National Instruments) was used to write scripts controlling the speed and sequence of the pumps.

**Figure 2:**
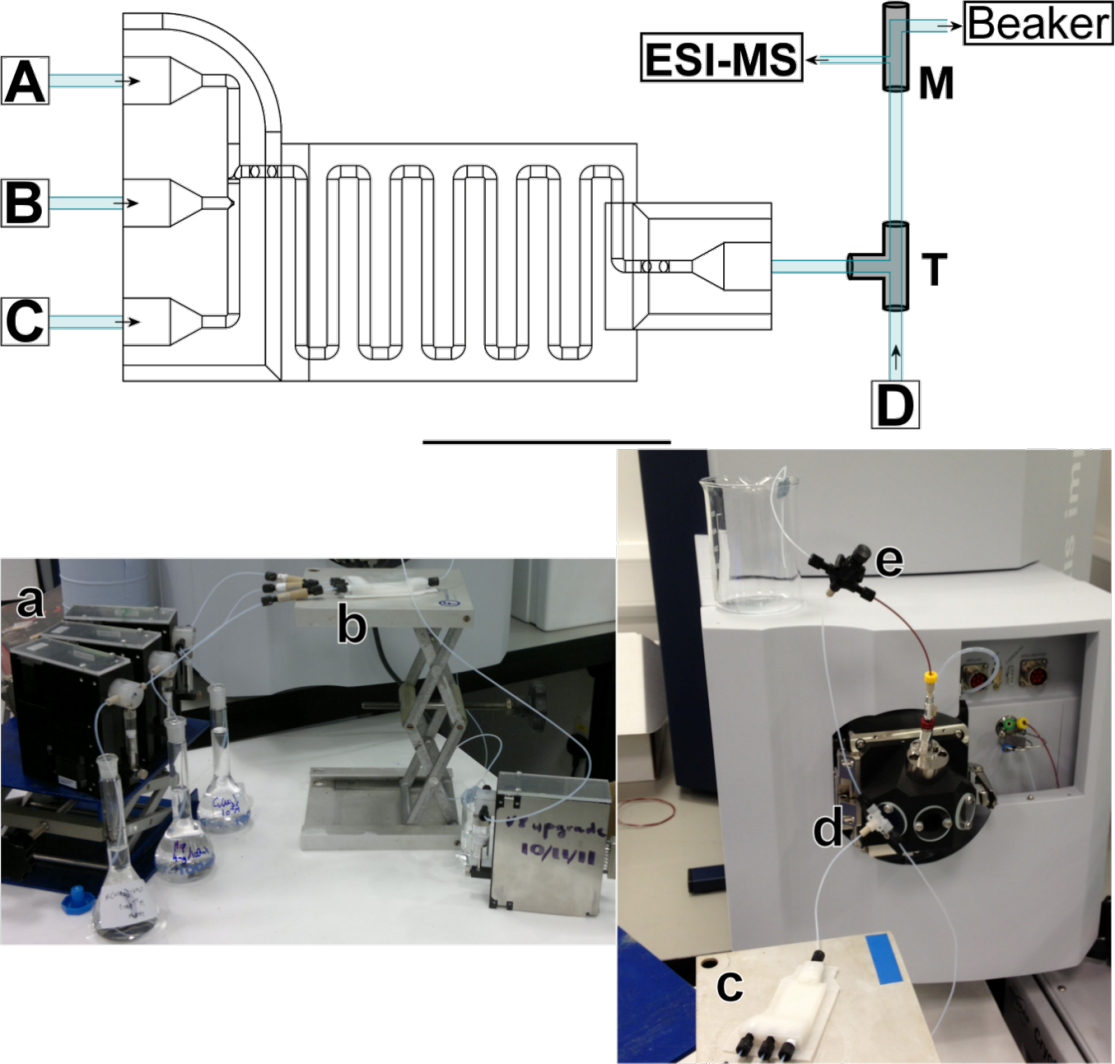
Top: A schematic overview of the device setup. The three inlets were each connected to a syringe pump, which were connected to stock solutions of the required starting materials or MeOH. The outlet is directly connected to a T-piece, where it mixes with a stream of MeOH for dilution. This is followed by the splitting step, where a PEEK microsplitter valve is used to split the stream so that only a suitable flow-rate reaches the ESI-MS. The parallel stream allows for collection of the reaction product. Bottom: Photograph of the device setup and connection to the mass spectrometer (where a = Tricontinent C-3000 syringe pumps; b = screw fittings; c = 3D printed device; d = T-piece; e = PEEK microsplitter valve).

To control the flow into the device and the overall residence time, syringe pumps were connected to the device through the standard plastic screw fittings and 1/16 inch (1.6 mm) tubing. The outlet was connected to a T-piece to allow for a dilution step to make the sample concentration suitable for ESI-MS analysis, and a PEEK microsplitter valve, which split the flow so that a section was directed to the collection point, whilst the remaining flow was directed to the in-line ESI-MS. An overview of the setup is presented in Figure 2.

### Chemistry

The ttop ligand was synthesised according to a reported literature procedure [25]. Solutions of ttop, Cu(NO_3_)_2_·6H_2_O and Ni(NO_3_)_2_·6H_2_O were all made up to 1 × 10^−4^ M in MeOH and were pumped into the devices via syringe pumps. The device setup was initially checked by running only ttop and MeOH, where ttop was run at a flow rate of 62.5 µL·min^−1^. The reactions between ttop and Cu(NO_3_)_2_·6H_2_O at different stoichiometries and alternating of the coordination of Cu(NO_3_)_2_·6H_2_O and Ni(NO_3_)_2_·6H_2_O to ttop, were then investigated. For the first set of experiments Cu(NO_3_)_2_·6H_2_O was pumped in through inlet A, ttop via inlet B, and MeOH via inlet C and D (see Figure 2 and Table 1).

**Table 1:** An overview of the solutions and flow rates used for the reactions of ttop and Cu at different stoichiometry.

**Pump**	**Solution** (1 × 10^−4^ M MeOH)	**Flow rate** (µL·min^−1^)			
		1:2	1:3	1:5	1:15

**A**	Cu(NO_3_)_2_·6H_2_O	62.5	93.75	93.75	93.75
**B**	ttop	31.25	31.25	18.75	6.25
**C**	MeOH	12.5	12.5	12.5	12.5
**D**	MeOH	62.5	62.5	62.5	62.5

The supramolecular product obtained when ttop was reacted with alternating Cu(NO_3_)_2_·6H_2_O and Ni(NO_3_)_2_·6H_2_O solutions was also investigated. The device setup was the same as for the first experiment, with the only difference being that Ni(NO_3_)_2_·6H_2_O was pumped in via inlet C, with pump A and C alternating (i.e., when pump A was active, pump C was idle, and vice versa) (see Table 2 for flow rates).

**Table 2:** An overview of the solutions and flow rates used for the reaction of ttop with Cu(NO_3_)_2_·6H_2_O and Ni(NO_3_)_2_·6H_2_O solutions.

**Pump**	**Solution** (1 × 10^−4^M MeOH)	**Flow rate** (µL·min^−1^)

**A**	Cu(NO_3_)_2_·6H_2_O	62.5
**B**	ttop	31.25
**C**	Ni(NO_3_)_2_·6H_2_O	62.5
**D**	MeOH	312.5

## Results and Discussion

The ttop ligand (**L**, C_24_H_24_N_6_) used in this investigation was produced by the reaction of *cis,trans*-1,3,5-triaminocyclohexane (*trans*-tach) with pyridine-2-carboxaldehyde (see Scheme 1). The ring-flip capability of the cyclohexane backbone allows two conformations, bis-equatorial mono-axial and bis-axial mono-equatorial. In the presence of a metal salt, the energetically favoured ring-flipped “closed” bis-axial monoequatorial conformer is retained and can be observed in the presented complexes **1** and **2**: [Cu(C_24_H_24_N_6_)(NO_3_)]^+^ (**1**) and [Ni(C_24_H_24_N_6_)(NO_3_)]^+^ (**2**). An axial tetradentate site (created from the N atoms of the axial pyridines and the associated imines) and an equatorial bidentate site (created from the two N atoms of the *trans* ttop arm) are formed in the favoured conformation, with the tetradentate site always being filled first due to it being the most energetically favoured site for metal ion coordination.

**Figure 3:**
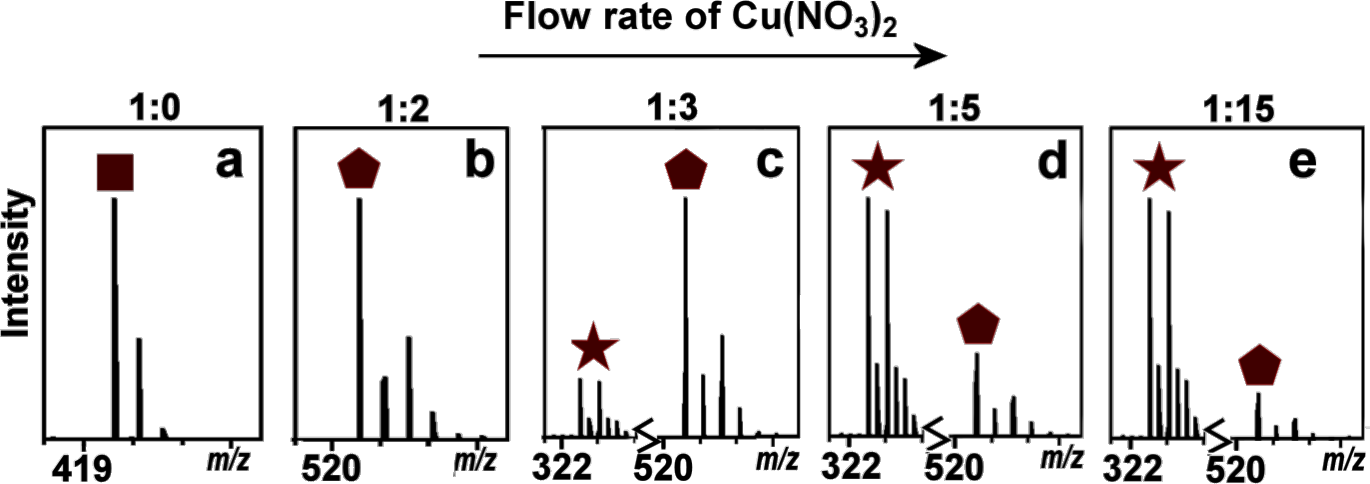
ESI-MS isotope patterns of ttop + Na (square), *m*/*z* 419.2, [Cu(C_24_H_24_N_6_)(NO_3_)]^+^ (**1**, pentagon), *m*/*z* 521.1, and [Cu_2_(C_24_H_24_N_6_)(NO_3_)_2_]^2+^ (**3**, star), *m*/*z* 323.0, showing how an increase in flow rate from the pump containing Cu(NO_3_)_2_·6H_2_O changes the stoichiometry of the complex from 1 ttop:1 Cu(NO_3_)_2_ to 1 ttop:2 Cu(NO_3_)_2_ (where a = MS of ttop; b = 1 ttop: 2 Cu(NO_3_)_2_·6H_2_O; c = 1 ttop: 3 Cu(NO_3_)_2_·6H_2_O; d = 1 ttop:5 Cu(NO_3_)_2_·6H_2_O; e = 1 ttop:15 Cu(NO_3_)_2_·6(H_2_O)) (for the full spectra see Supporting Information File 1).

As mentioned previously, ttop (1 × 10^−4^ M) was first introduced through the device (with methanol in the three remaining active pumps) at a flow rate of 62.5 µL·min^−1^ with the product stream then split after a dilution step to allow continuous parallel collection and analysis. When the product arrived at the mass spectrometer a mass spectrum with isotope envelopes at *m*/*z* 397.2 and 419.2 for the species [C_24_H_25_N_6_]^+^ (**L** + H) and [C_24_H_25_N_6_Na]^+^ (**L** + Na), respectively, was observed (Figure 3a). Cu(NO_3_)_2_·6H_2_O (1 × 10^−4^ M) was then introduced to syringe pump A and complexed with ttop at flow rates of 31.25 µL·min^−1^ and 62.5 µL·min^−1^ for ttop and Cu(NO_3_)_2_·6H_2_O, respectively (1 equiv ttop:2 equiv Cu(NO_3_)_2_·6H_2_O). This complexation reaction lead to the formation and observation of the species [Cu(C_24_H_24_N_6_)(NO_3_)]^+^ (**2**), *m*/*z* 521.1 (Figure 3b). When the flow rate of Cu(NO_3_)_2_·6H_2_O was increased from 62.5 µL·min^−1^ to 93.75 µL·min^−1^ (1 equiv ttop:3 equiv Cu(NO_3_)_2_·6H_2_O) the Cu(NO_3_)_2_·6H_2_O flow rate was increased by such an amount that the 1:2 stoichiometric species [Cu_2_(C_24_H_24_N_6_)(NO_3_)_2_]^2+^ (**3**) at *m*/*z* 323.0 could be seen to grow, but with the abundance still being lower than that of the [Cu(C_24_H_24_N_6_)(NO_3_)]^+^ (**2**) species (Figure 3c). The ttop flow rate was then decreased to 18.75 µL·min^−1^ (1 equiv ttop:5 equiv Cu(NO_3_)_2_·6H_2_O) to give a reaction mixture in which there was a higher abundance of [Cu_2_(C_24_H_24_N_6_)(NO_3_)_2_]^2+^ (**3**) compared to [Cu(C_24_H_24_N_6_)(NO_3_)]^+^ (**2**) (Figure 3d). The rate was then decreased further to a rate at which the ttop pump ran at 6.25 µL·min^−1^ (1 equiv ttop:15 equiv Cu(NO_3_)_2_·6H_2_O) to produce predominantly the [Cu_2_(C_24_H_24_N_6_)(NO_3_)_2_]^2+^ (**3**) species (Figure 3e, a low intensity residual peak of the monocopper complex is also observed here).

In-house LabView scripts were then utilised to vary the metal-salt coordination. Here scripts were developed to allow the careful control of oscillating reaction mixtures (cycles) where pumps A and C were alternated to allow complexation of Ni(NO_3_)_2_·6H_2_O and Cu(NO_3_)_2_·6H_2_O, respectively. The experiment was set up as shown in Figure 2 with Cu(NO_3_)_2_·6H_2_O (1 × 10^−4^ M) in pump A, ttop (1 × 10^−4^ M) in pump B and Ni(NO_3_)_2_·6H_2_O (1 × 10^−4^ M) in pump C. Pump D contained MeOH for an additional diluting stage to decrease the mixture concentration before introduction to the mass spectrometer.

**Figure 4:**
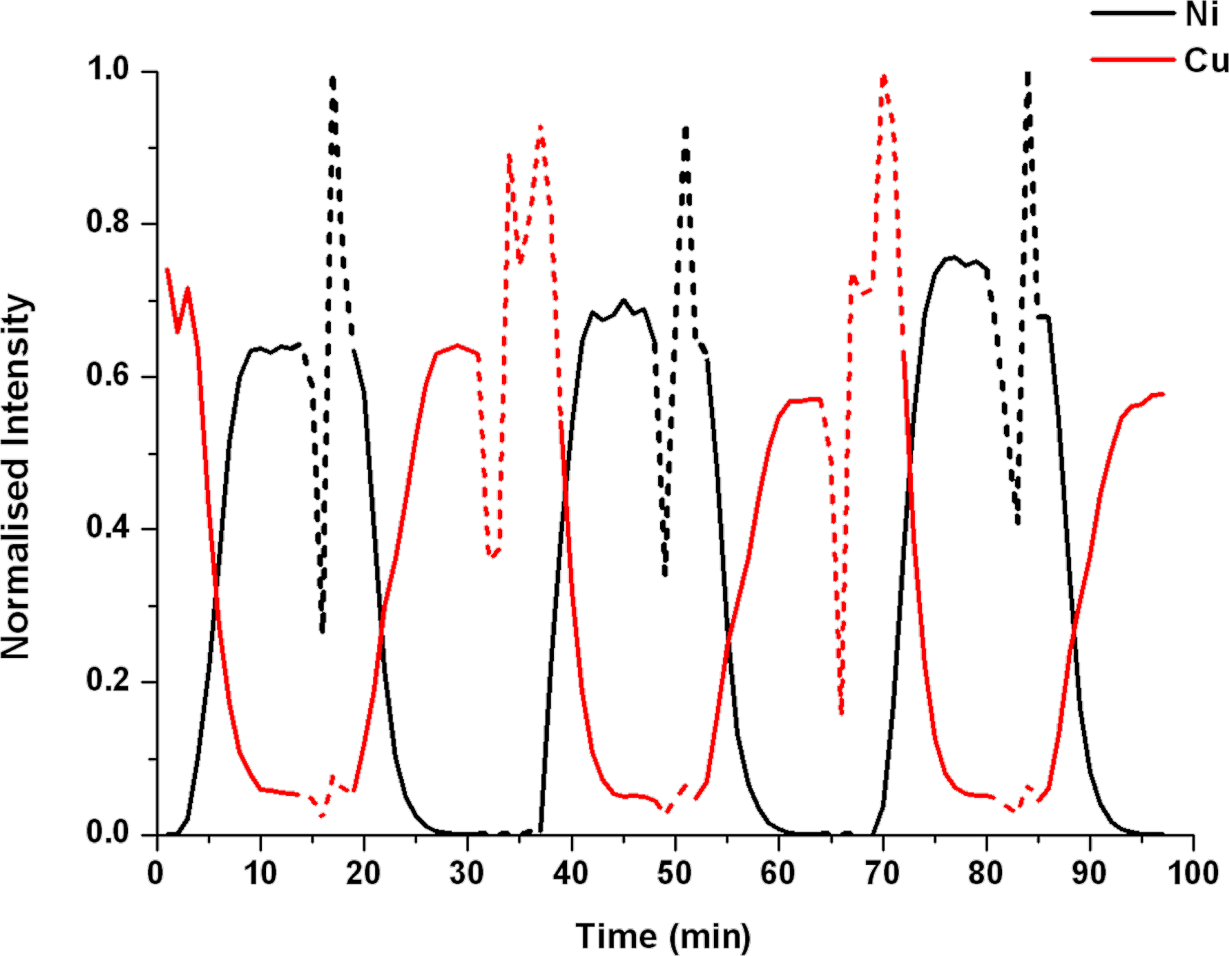
Normalised intensity plotted against time showing the change in intensity of [Ni(C_24_H_24_N_6_)(NO_3_)]^+^ (**2**), *m*/*z* 516.1, (black) and [Cu(C_24_H_24_N_6_)(NO_3_)]^+^ (**1**), *m*/*z* 521.1, (red) over five pump cycles. (The dotted lines correspond to the instability in the intensity of the species due to the pumps refilling, and thus, the system is not at equilibrium).

The oscillation was carried out by firstly filling pumps B and C at the same rate, with an outlet flow rate at pump C (containing Ni(NO_3_)_2_·6H_2_O) of 62.5 µL·min**^−^**^1^ and pump B (containing ttop) at a flow rate of 31.25 µL·min**^−^**^1^. The reaction solutions were then mixed together in the device to form the complex [Ni(C_24_H_24_N_6_)(NO_3_)]^+^ (**2**), *m*/*z* 516.1. The residence time of the device (the time that the reactants were allowed to react within the device) was then calculated from when the species of interest was formed; approximately 5 min. (Please note that the first two cycles were omitted due to the device channels being partially unfilled and so gave an unstable and unrealistic signal at the mass spectrometer, and the data was continuously recorded over seven cycles). The next cycle was initiated after 32 min, where the activated pumps were switched from Ni(NO_3_)_2_·6H_2_O and ttop to Cu(NO_3_)_2_·6H_2_O and ttop, to form the species Cu(C_24_H_24_N_6_)(NO_3_)]^+^ (**1**), *m*/*z* 521.1, which can be observed in the mass spectrometry data approximately 5 min after initiation. Analysis of the continuous parallel ESI-MS data collected showed both the species [Ni(C_24_H_24_N_6_)(NO_3_)]^+^ (**2**), *m*/*z* 516.1, and [Cu(C_24_H_24_N_6_)(NO_3_)]^+^ (**1**), *m*/*z* 521.1, present in the reaction mixture for a further 10 min until only the [Cu(C_24_H_24_N_6_)(NO_3_)]^+^ (**1**), *m*/*z* 521.1, species was present and the system was at equilibrium. This oscillation pattern was then repeated for a further three cycles (see Figure 4). From the MS data and Figure 4 it can be seen that after approximately 10 min into each cycle the reaction reached a steady state, and so from the stream at this time a sample of the complex can be collected for further analysis and/or reactions. Due to the pumps having to refill, a drop-off in intensity was observed in the mass spectrum after 16 min in each cycle. This drop-off was then followed by an increase in intensity of the complex in question (dotted lines in Figure 4), then a rapid decrease in intensity accompanied by the formation and increase in intensity of the next complex. This intensity instability (dotted lines in Figure 4) will need to be further researched to provide an answer as to why the intensity fluctuates as it does. Further work using a peristaltic type system, where refilling would be continuous, would remove the intensity instability caused by the pumps restarting.

For the relative intensity of the [Ni(C_24_H_24_N_6_)(NO_3_)]^+^ (**2**), *m*/*z* 516.1 and [Cu(C_24_H_24_N_6_)(NO_3_)]^+^ (**1**), *m*/*z* 521.1 species see Supporting Information File 1.

## Conclusion

By carrying out a number of complementary supramolecular chemical reactions in bespoke 3D-printed reactionware we have shown their high potential and configurability. The tailored milliscale reactionware, with the appropriate number of inlets, outlets and reactor length, was successfully utilised as a fluidic device. The design of the device allowed standard screw fittings to be employed for connecting the device to the desired pumps and other fittings, in this instance a T-piece for dilution and a PEEK microsplitter valve device for splitting the product stream. This design, utilising the addition of screw fittings, allows for better connections, seals, and easier reuse of the devices, compared to previously published procedures [4].

We have presented, for the first time, an example of a configurable, real-time, continuous parallel-flow technique using ESI-MS as an analytical tool and a 3D printed device as the reaction vessel. The stream splitting allows direct interfacing of the outlet stream with in-line ESI-MS, with flow rates fast enough for ESI-MS and sample collection.

Supramolecular chemistry was carried out to emphasise the versatility of the reactionware setup. Changing the flow rate of the reactants allows the successful change in product stoichiometry from 1:1 to 2:1, as confirmed by in-line ESI-MS. The same reactionware setup can be used to switch between two ttop complexes, where the Cu and Ni salts were alternately pumped through the device, with ttop remaining constant. Real-time, continuous ESI-MS was successfully used to observe the reaction and the oscillation between the two salt complexes with a simultaneous collection of the product.

In the near future we aim to further expand and improve the reactionware, for example, by including heat exchangers. For the in-line ESI-MS setup we intend to further utilise the split product stream by including other in-line techniques, such as UV–vis and IR spectroscopy. To further develop the 3D printing technology we are currently investigating the use of various new print materials, for example active functional materials, to highlight the versatility and configurability of 3D printing and 3D-printed reactionware. We are also designing new geometries that will provide reaction conditions currently difficult to achieve with traditional glassware/millifluidic techniques.

## Supporting Information

File 1 Additional experimental data.

File 2 Device design as a compressed STL file (editable, e.g., with Autodesk123D^®^).
