# Supplementary material for: Continuous parallel ESI-MS analysis of reactions carried out in a bespoke 3D printed device
Source: Beilstein J Nanotechnol. 2013 Apr 29;4:285–91. doi: 10.3762/bjnano.4.31 (PMC3678396; doi:10.3762/bjnano.4.31)
Supplement: File 1 — Additional experimental data. [file Beilstein_J_Nanotechnol-04-285-s001.pdf]

Supporting Information

for

Continuous parallel ESI-MS analysis of reactions carried out in a bespoke 3D printed device

Jennifer S. Mathieson, Mali H. Rosnes, Victor Sans, Philip J. Kitson and Leroy Cronin*

Address: School of Chemistry, University of Glasgow, G12 8QQ, United Kingdom

Email: Leroy Cronin* - Lee.Cronin@glasgow.ac.uk

* Corresponding author

Additional experimental data

Table of Content

1 General Experimental Remarks........S2

1.1 Design Software........S2

1.2 Device Setup........S2

1.3 ESI-MS........S4

2 ESI-MS Data........S5

1 General experimental remarks

All chemical reagents and solvents were purchased from Sigma Aldrich and used without further purification.

1.1 Design software: The 3D-printed device used in this work was designed on the freely distributed 3D CAD software Autodesk123D® (http://www.123dapp.com/) although any 3D modelling/CAD software with the ability to export models in an STL file (Supporting Information File 2) format would suffice for this, and there are a number of suitable alternative free/open source candidates available on the internet. The device design was exported as an STL file (available from the authors), which was then interpreted by Bits from Bytes Axon 2 software, which produces a 3D printer instruction file (BFB file), which was subsequently transferred to the 3DTouch™ 3D printer. The printing was conducted in a layer-by-layer fashion by the 3DTouch™ printer, and the device was printed using polypropylene (PP) and fitted with standard PTFE 1/16" (1.6 mm) OD tubing and standard screw connectors. The inlet tubing was subsequently connected to the pumps, whilst the outlet was fitted with a T-device for dilution and a polyether ether ketone (PEEK) microsplitter valve with PEEK tubing to control the flow to the ESI-MS.

1.2 Device setup: The overall dimensions of the device are 46.5 × 80 mm. It takes five hours to print, and the inner path of the device is about 1.5 mm in diameter. The total internal capacity of the device is roughly 0.65 mL, and the actual reaction capacity is about 0.57 mL. The printed device weighs about 20 g and can be valued at approximately US$ 0.40 (€ 0.30). The total cost of the PEEK accessories was US$ 230 (€ 180) and the Tricontinent C-3000 syringe pumps with the associated

hardware cost US$ 1000 (€ 770) per pump. Therefore the total cost was approximately US$ 4200 (€ 3300).

All solutions were pumped by means of Tricontinent C-3000 syringe pumps equipped with 1 mL syringes for the starting materials, and with a 5 mL syringe for the dilution step. An in-house-developed LabView application was employed to program the pumps to deliver the desired flow rates.

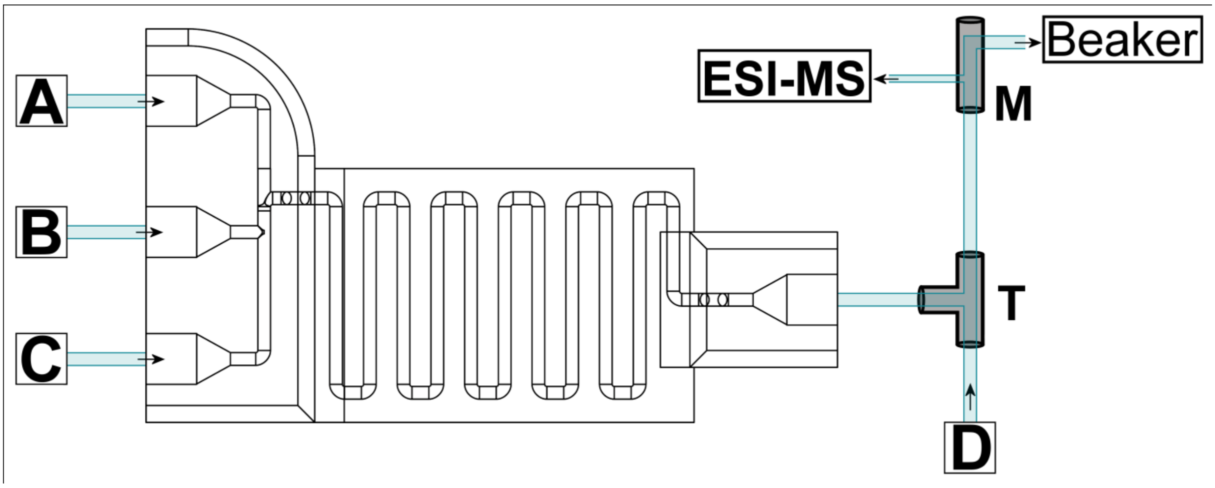

Figure S1: The device setup, where the numbers denote the four pumps used, where pump A, B and C have 1 mL syringes, and pump D has a 5 mL syringe. The pale blue channels represent the tubing, the T denotes the T-piece where the dilution takes place, whilst M denotes the PEEK microsplitter valve, which splits the flow so that only the required amount will reach the ESI-MS. Check-valves were fitted between the pumps and inlet A–C, about 1.5 cm from the device, to avoid backflow and diffusion when the pumps were idle.

As seen in **Figure S2**, it is evident that there is no backflow or diffusion issue for inlet C when only pumps A and B are moving as the solution is still colourless between the inlet and the check-valve for this inlet. On closer inspection it can be seen that a blue area is in front of the purple area within the reactor device. This is because pump A

runs twice as fast as pump B, and therefore the area at the front is only Methylene Blue. To avoid this problem during our experiments we ignored the first two runs, allowing the device to be properly filled first.

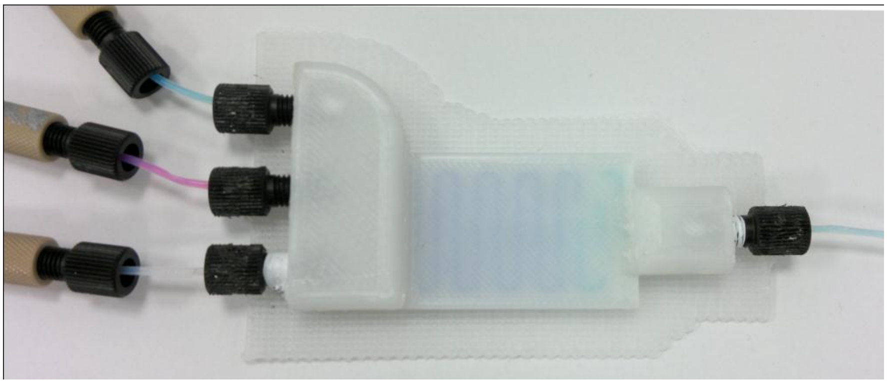

Figure S2: A photograph of the device with Methylene blue in inlet A, Rhodamine B in inlet B, and pump C idle. It can be seen how the blue and red colours mix from the resulting purple colour, behind the blue solution. The first two runs are ignored to avoid the initial solution coming from only the fastest pump.

1.3 ESI-MS: The experiments were carried out at 180 °C at concentrations of the complex in the region of 10^{-4} and 10^{-5} mol·L^{-1} in methanol by using a Bruker MaXis Impact instrument. The calibration solution used was Agilent ESI-L low concentration tuning mix solution, Part No. G1969-85000, enabling calibration between approximately 50 *m*/*z* and 2000 *m*/*z*. Samples were dissolved in CH_3OH (10^{-4} M) and introduced into the MS at a dry gas temperature of 180 °C. The ion polarity for all MS scans recorded was positive, with the voltage of the capillary tip set at 4500 V, end plate offset at −500 V, funnel 1 RF at 400 Vpp and funnel 2 RF at 400 Vpp, hexapole RF at 200 Vpp, ion energy 5.0 eV, collision energy at 5 eV, collision cell RF at 200 Vpp, transfer time at 100.0 µs, and the pre-pulse storage time at 10.0 µs. Each spectrum was collected for 100 min.

2 ESI-MS data

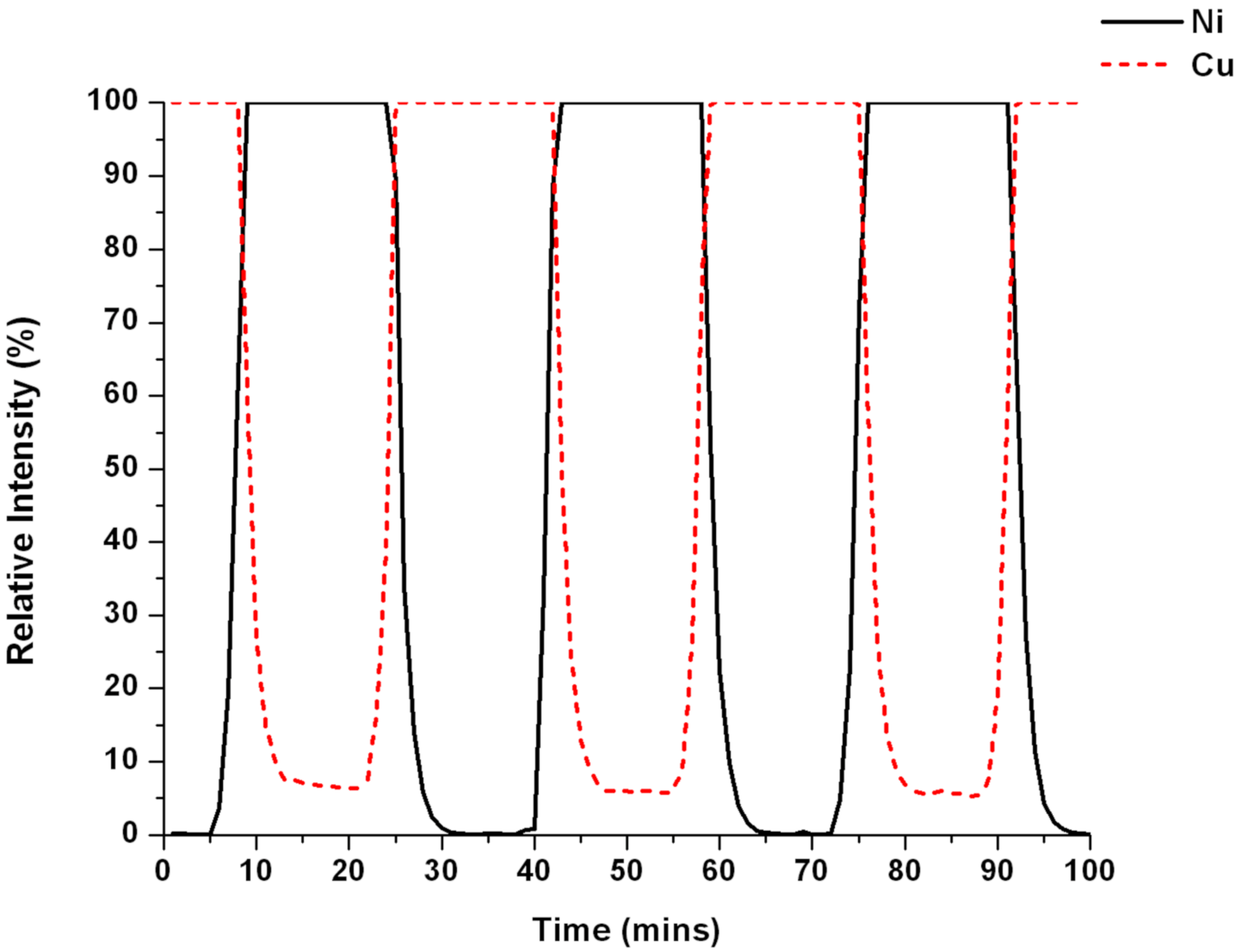

Figure S3: Relative intensity of the $[Ni(C_{24}H_{24}N_6)(NO_3)]^+$ (**2**), *m*/*z* 516.1, (black) and $[Cu(C_{24}H_{24}N_6)(NO_3)]^+$ (**1**), *m*/*z* 521.1, (red) species over time showing five oscillation cycles.

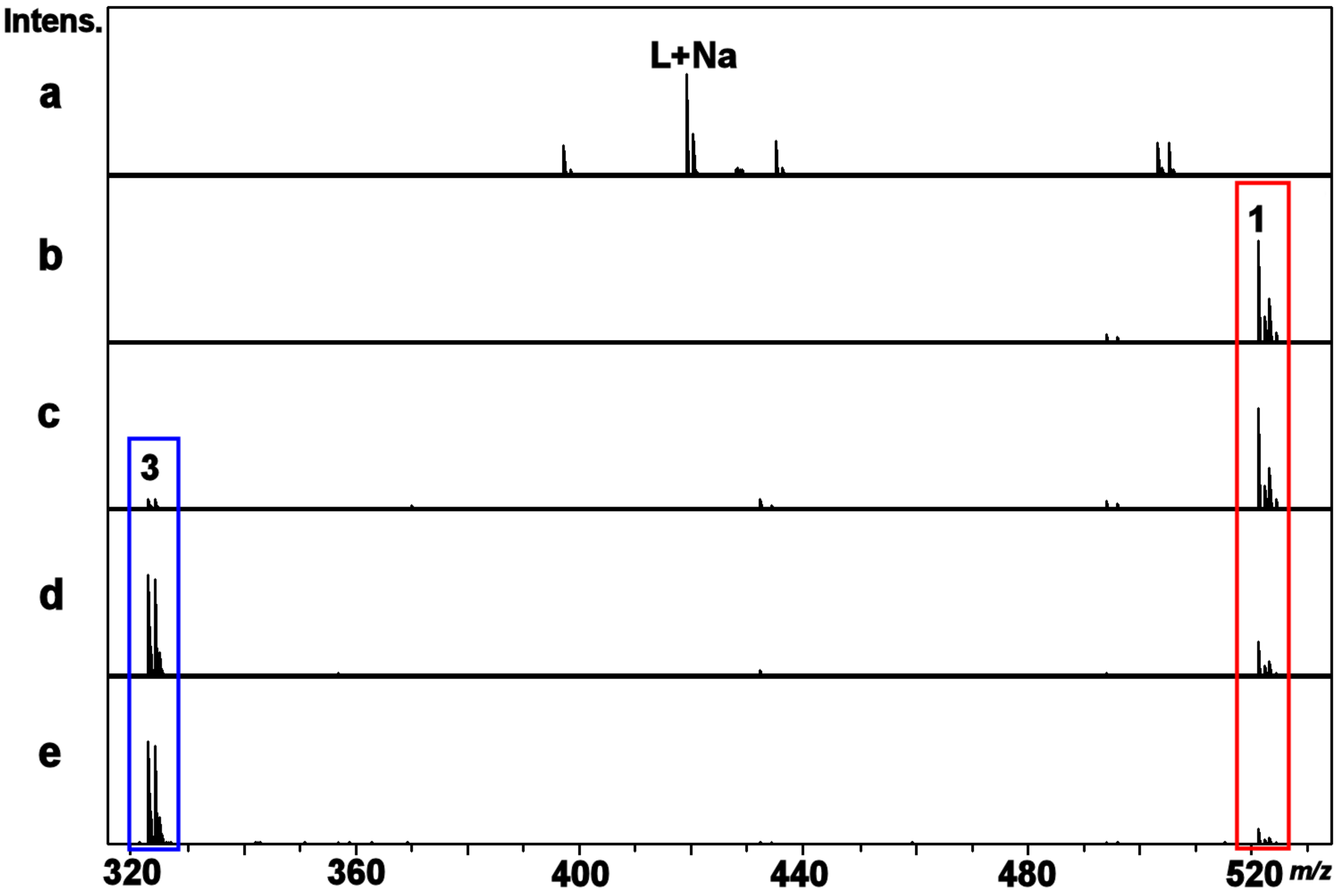

Figure S4: MS spectra showing the change in stoichiometry from $[Cu(C_{24}H_{24}N_6)(NO_3)]^+$ (**1**), *m/z* 521.1 to $[Cu_2(C_{24}H_{24}N_6)(NO_3)_2]^{2+}$ (**3**), *m/z* 323.0 when the flow rate of the $Cu(NO_3)_2{\cdot}6H_2O$ was increased (where a = MS of ttop; b = 1 ttop:2 $Cu(NO_3)_2{\cdot}6H_2O$; c = 1 ttop:3 $Cu(NO_3)_2{\cdot}6H_2O$; d = 1 ttop:5 $Cu(NO_3)_2{\cdot}6H_2O$; e = 1 ttop:15 $Cu(NO_3)_2{\cdot}6H_2O$).
